# Ataxia-telangiectasia-like disorder in a family deficient for MRE11A, caused by a *MRE11* variant

**DOI:** 10.1212/NXG.0000000000000295

**Published:** 2018-12-03

**Authors:** Maryam Sedghi, Mehri Salari, Ali-Reza Moslemi, Ariana Kariminejad, Mark Davis, Hayley Goullée, Björn Olsson, Nigel Laing, Homa Tajsharghi

**Affiliations:** From the Medical Genetics Laboratory (M. Sedghi), Alzahra University Hospital, Isfahan University of Medical Sciences, Isfahan, Iran; Department of Neurology (M. Salari), Shahid Beheshti University of Medical Science, Tehran, Iran; Department of Pathology (A.-R.M.), University of Gothenburg, Sahlgrenska University Hospital, Sweden; Kariminejad-Najmabadi Pathology & Genetics Center (A.K.), Tehran, Iran; Department of Diagnostic Genomics (M.D.), Pathwest, QEII Medical Centre; Centre for Medical Research (H.G., N.L., H.T.), The University of Western Australia and the Harry Perkins Institute for Medical Research, Nedlands, Australia; School of Bioscience (B.O.), University of Skovde; and Division Biomedicine (H.T.), School of Health and Education, University of Skovde, Sweden.

## Abstract

**Objective:**

We report 3 siblings with the characteristic features of ataxia-telangiectasia-like disorder associated with a homozygous *MRE11* synonymous variant causing nonsense-mediated mRNA decay (NMD) and MRE11A deficiency.

**Methods:**

Clinical assessments, next-generation sequencing, transcript and immunohistochemistry analyses were performed.

**Results:**

The patients presented with poor balance, developmental delay during the first year of age, and suffered from intellectual disability from early childhood. They showed oculomotor apraxia, slurred and explosive speech, limb and gait ataxia, exaggerated deep tendon reflex, dystonic posture, and mirror movement in their hands. They developed mild cognitive abilities. Brain MRI in the index case revealed cerebellar atrophy. Next-generation sequencing revealed a homozygous synonymous variant in *MRE11* (c.657C>T, p.Asn219=) that we show affects splicing. A complete absence of *MRE11* transcripts in the index case suggested NMD and immunohistochemistry confirmed the absence of a stable protein.

**Conclusions:**

Despite the critical role of MRE11A in double-strand break repair and its contribution to the Mre11/Rad50/Nbs1 complex, the absence of MRE11A is compatible with life.

Neurological defects, including microcephaly, ataxia or neurodegeneration, are a hallmark for autosomal recessive mutations in individual genes encoding components of the Mre11/Rad50/Nbs1 (MRN) complex. Mutations in the NBS1 encoding gene, *NBN*, are associated with Nijmegen breakage syndrome (NBS) (OMIM# 251260) characterized by microcephaly, immunodeficiency, growth and intellectual disability, radiosensitivity, and cancer predisposition.^1–3^ Mutations in RAD50 gene *RAD50* are related to NBS-like disorder (NBSLD) (OMIM# 613078).^4^ The clinical features of patients with NBSLD are very similar to those with NBN, including microcephaly and intellectual disability but not infections, immunodeficiency, or cancer predisposition. Mutations in MRE11A homolog, double-strand break repair nuclease gene *MRE11* are associated with a very rare chromosomal breakage syndrome (OMIM# 604391). The clinical features are characterized by progressive cerebellar degeneration and ionizing radiation hypersensitivity, similar to the ataxia telangiectasia (OMIM# 208900) caused by mutations in *ATM*, encoding ataxia telangiectasia mutated (ATM).^5,6^ The neurologic features have a later onset, slower progression and it is referred to as ataxia-telangiectasia-like disorder (ATLD) (OMIM# 604391).^5,7,8^ Unlike patients with ataxia telangiectasia, however, patients with ATLD show no telangiectasia or obvious immunodeficiency.^7–9^ In 1999, first association of ATLD with mutations in *MRE11* was reported,^5^ followed by additional families with clinical features of ATLD^9–16^ or NBSLD.^17^

Here, we report a family with characteristic features of ATLD associated with a novel homozygous apparently synonymous variant in exon 7 of *MRE11* that ablates normal splicing, induces nonsense-mediated mRNA decay (NMD), and deficiency of MRE11A protein.

## Methods

### Standard protocol approvals, registrations, and patient consents

The study was approved by the ethical standards of the relevant institutional review board, the Ethics Review Committee in the Gothenburg Region (Dn1: 842-14), and the Human Research Ethics Committee of the University of Western Australia. Informed consent was obtained from the parents included in this study after appropriate genetic counseling. Blood samples were obtained from patients and their parents.

### Clinical evaluation

Medical history was obtained and physical examination was performed as part of routine clinical workup.

### Genetic analysis

Next-generation sequencing (NGS) (a targeted neuromuscular sub-exomic sequencing [NSES] panel and/or whole exome sequencing [WES]) was performed on patients' DNA. Confirmatory bidirectional Sanger sequencing was performed in the patients and all available unaffected family members (e-Methods, links.lww.com/NXG/A128).

### Analyses of muscle biopsy

A muscle biopsy was obtained in the index patient (V:2). Morphologic and histochemical analyses of paraffin-embedded muscle tissue were performed according to standard protocols. Sections of skeletal muscle tissue were processed for transcript, histologic and immunohistochemical assessments (e-Methods, links.lww.com/NXG/A128).

### Transcript analysis

To analyze the impact of the c.657C>T variant in splicing efficiency of *MRE11* exon 7 in the index case (V:2), polymerase chain reaction (PCR) was performed on complementary DNA with primer pairs covering exon 1 through 8 (e-Methods, links.lww.com/NXG/A128).

### Chromosomal assay

Peripheral blood samples were obtained from 9 individuals of the family (III:1, IV:1, IV:2, IV:8, IV:9, V:1, V:2, V:3, and V:4). Chromosomal breakage tests using mitomycin C (MMC) induction in cultures was carried out according to standard protocols. Metaphases were stained and scored for spontaneous chromosomal anomalies. Twenty metaphase spreads were studied from routine culture, 100 spreads from culture were prepared with the addition of 2 concentrations of MMC. These were compared with 100 spreads from age-matched normal controls. Unfortunately, no additional tissue was available for assessment of the effect of *MRE11* c.657C>T variant on sensitivity to ionizing radiation and the impact on NBS1 and RAD50 expression levels or the entire MRN complex.

## Data availability

Data not published within the article are available online in supplemental material (links.lww.com/NXG/A128).

## Results

### Clinical characteristics of patients

**Figure 1:**
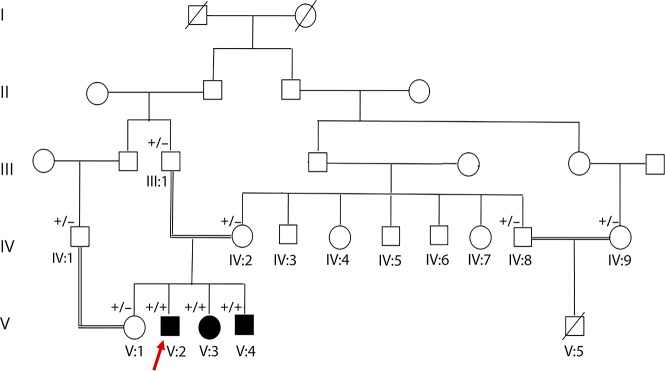
Pedigree of the family Pedigree and recessive inheritance of *MRE11*. In the pedigree, squares represent males; circles, females; open symbols, unaffected family members; and slash, deceased. The affected individuals are represented with shaded symbols. Arrow indicates the proband in the family (V:2). +/− indicates heterozygous presence of the variant; +/+ indicates homozygous appearance of the variant.

**Figure 2:**
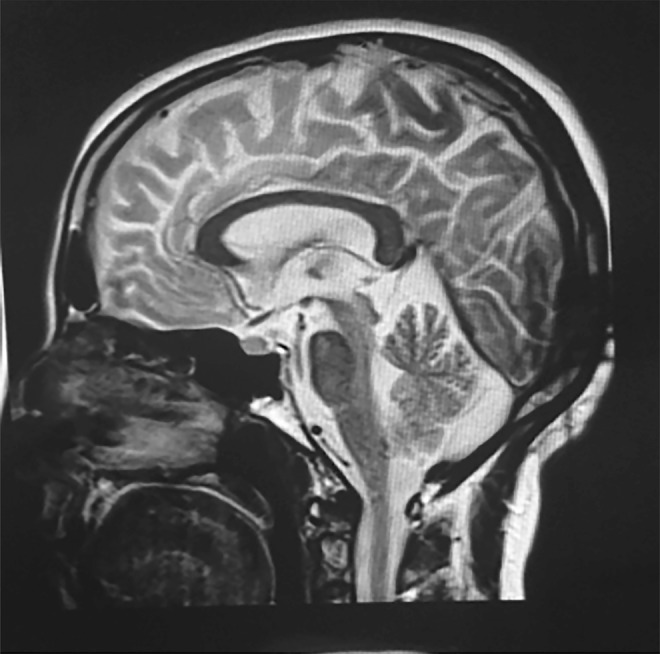
Sagittal T1-weighted MRI of brain Brain MRI from the index case (V:2) shows significant atrophy of the cerebellar vermis. The brainstem is relatively preserved.

Three affected siblings were born to an apparently healthy consanguineous couple (figure 1). Their clinical presentations were consistent with the characteristic features of ATLD. The affected individuals presented with developmental delay during the 1st year of age. They developed poor balance and suffered from mild intellectual disability from early childhood. The symptoms progressed gradually. Chromosomal study revealed a normal 46, XY (V:2 and V:4) or 46, XX (V:3) pattern. There was no history of severe or recurrent infections in the index case or in his 2 affected siblings. Follow-up at 23 (V:2), 20 (V:3), and 18 (V:4) years of age revealed oculomotor apraxia, slurred and explosive speech, limb and gait ataxia, exaggerated deep tendon reflexes (+3), dystonic posture, mirror movement in their hands, and poor balance (video 1). They had no sensory deficits. IQ was between 50 and 69 in all affected siblings. There was no facial dysmorphism (video 1); however, measurements including height, weight, and head circumference were below the 3rd percentile in all 3 affected siblings. Systemic examinations of the affected siblings were normal without any evidence of skeletal deformity or skin lesions. The laboratory evaluation, including thyroid function, liver function test, and alpha-fetoprotein, revealed levels in the reference range. Echocardiography of the youngest sibling (V:4) at 16 years of age was unremarkable. Nerve conduction velocity and EMG recording in the index case (V:2) did not show any neuropathic or myopathic features. Brain MRI in the index case (V:2) at 21 years of age revealed cerebellar atrophy (figure 2). The cousin of the affected siblings (V:5) (figure 1) was a boy with Down syndrome, who died at 6 months of age because of tetralogy of Fallot. However, he was not clinically evaluated for ATLD. There is no family history of cancer.

10.1212/000295_Video_1 Video 1 The proband (V:2) shows no dysmorphic facial features, however poor balance is evident.Download Supplementary Video 1 via http://dx.doi.org/10.1212/000295_Video_1

### Genetic findings

**Figure 3:**
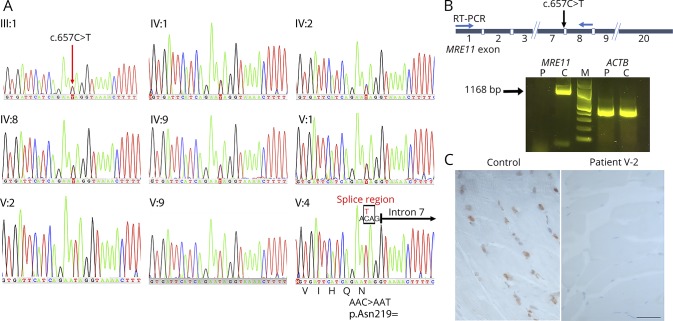
Genetic findings and expression analysis of MRE11A at transcript and protein levels (A) Sanger sequence analysis demonstrates the segregation of the MRE11 c.657C>T variant in the family. The unaffected parents (III:1 and (IV:2) and additional family members (IV:1, IV:8, IV:9, and V:1) are heterozygous for the MRE11 c.657C>T variant. The affected individuals for whom DNA was available (V:2, V:3, and V:4) are homozygous for the variant. The variant does not lead to alteration of asparagine amino acid (AAC>AAT, p.Asn219=). The variant is located in splice region (indicated by a box) changing the nucleotide of the 3′ end of exon 7 (ENST00000323929.7). The 5′ end of intron 7 is shown by an arrow. (B) reverse-transcriptase polymerase chain reaction analysis of RNA from skeletal muscle tissues of the index case (V:2) and a control. Expression analysis of fragment covering exon 1 through 8 coding regions of MRE11 (1168 bp) at the transcript level in the patient and a control indicated the absence of MRE11 transcript in the patient. Amplification of ACTB served as quality control of complementary DNA. (C) Immunostaining of MRE11A in skeletal muscle biopsy specimen from the index patient (V:2) indicates no nucleoplasm expression, whereas the control muscle specimen shows positive immunostaining of MRE11A localized to nucleoplasm. Scale bar, 50 µm.

Data from NGS of DNA from 2 affected (V:2 and V:3) and 1 unaffected family members (V:1) were analyzed. Targeted sequencing of 336 known neurogenic disease genes, including 32 ataxia-associated genes in DNA from the index case V:2 identified a *MRE11* variant changing the nucleotide 3 bases from the 3′ end of exon 7 (c.657C>T, rs775017362) in the homozygous state. The variant did not alter the coded amino acid (AAC>AAT, p.Asn219=). No rare, likely pathogenic heterozygous or homozygous variants were identified in other neurogenetic- or ataxia-associated genes included in the targeted panel, including *ATM*, *APTX*, or *SETX*. Simultaneous WES on DNA samples from individuals V:1, V:2, and V:3 to look for variants in novel disease genes was performed. The filtering strategy of initially concentrating on homozygous coding variants in known neurogenetic disease genes, selected based on variant databases Human Genome Mutation Database and ClinVar, and most recent literature allowed the identification of only the same homozygous c.657C>T variant in *MRE11* as identified by NSES analysis. The *MRE11* variant was identified in the heterozygous state in individual V:1 (figure 3A). The c.657C>T substitution was present at very low frequency in the genome Aggregation Databases (1/245,540 alleles). In silico analysis predicted the *MRE11* variant to be possibly disease causing, presumably identified as potentially affecting splicing (MutationTaster, mutationtaster.org/). In silico prediction with the SpliceAid2 (introni.it/spliceaid.html) indicated that the variant was located in the splicing regulatory sequences, suggesting altered splicing factors binding sites. In addition, in silico prediction with the Human Splicing Finder version 3.0 and MaxEntScan suggested that the variant has a deleterious effect on the gene and creates an exonic splice site loss, leading to a mRNA frameshift and subsequently to a premature termination codon.

The appearance of the *MRE11* variant was examined in all available family members by sequencing analysis (figure 3A). Sanger sequencing confirmed segregation of the variant with the disease phenotype. The 3 affected individuals (V:2, V:3, and V:4) were homozygous for the c.657C>T variant; the unaffected parents (III:1 and IV:2) and 1 unaffected sibling (V:1) were heterozygous carriers of the *MRE11* variant. In addition, the unaffected individuals IV:1, IV:8, and IV:9 were heterozygous for the *MRE11* variant (figure 3A).

### Transcript analysis

Muscle biopsy from the index case (V:2) was available for testing the effects of the homozygous c.657C>T *MRE11* exon 7 variant on transcript and protein levels. In silico prediction suggested splicing defect with a likely consequence of a premature termination codon. Accordingly, the reverse-transcriptase PCR analysis indicated no detectable expression levels of *MRE11* transcript in the index case (figure 3B), suggesting that this variant destabilizes the transcript by NMD.

### Histological and immunohistochemical analysis of skeletal muscle

Immunohistochemical analysis on the muscle biopsy from the index case (V:2) was performed to assess the nuclear expression of MRE11A protein. Skeletal muscle biopsies from 2 individuals without neurodegenerative disorders were used as controls. In contrast to control skeletal muscle biopsies showing detectable expression levels of MRE11A protein in the nucleoplasm, MRE11A immunohistochemistry using a polyclonal antibody showed no staining in the skeletal muscle biopsy from the index case (figure 3C), indicating lack of a stable truncated protein.

### Chromosomal aberration assay

Chromosomal breakage more than or equal to tenfold of control is clinically significant. Chromosomal breakage tests carried out for 9 individuals of the family (III:1, IV:1, IV:2, IV:8, IV:9, V:1, V:2, V:3, and V:4) indicated no significant structural alterations, such as chromosomal breaks, chromosomal translocations or gaps, when compared with the controls at 450–550 band resolution.

## Discussion

In this study, we describe a family with 3 affected siblings, at 23, 20, and 18 years of age, diagnosed with ATLD. Consistent with this diagnosis, the siblings developed progressive cerebellar ataxia, developmental delay, and mild intellectual disability but with absence of telangiectasia or facial dysmorphism and no history of severe infections, immunodeficiency, or cancer.

Next-generation sequencing revealed homozygosity in the affected individuals for the *MRE11* (c.657C>T, p.Asn219=) rs775017362 variant, which is present in population databases at frequencies compatible with recessive inheritance. The variant does not change the amino acid, but in silico analysis predicted that the variant would affect splicing efficiency, most likely resulting in exon 7 skipping, leading to a frameshift and a premature termination codon (p.Ser183Valfs*31). The homozygous variant could only be detected from the sequencing of genomic DNA. No *MRE11* alleles were revealed by transcript assessment, making it likely that this variant destabilizes the transcript by NMD. This correlated with immunolabeling findings, demonstrating no MRE11A staining in the skeletal muscle biopsy from the index case, suggesting the absence of a stable protein.

So far, several families with *MRE11* variants have been reported.^5,9–13,17^ A majority of reported variants in *MRE11*, homozygous or compound heterozygous splicing, nonsense or missense mutations, have been associated with a spectrum of clinical severity of ATLD^5,9–13^ (table). However, *MRE11* variants have been described in 2 unrelated Japanese patients with characteristic features of NBSLD,^17^ which is otherwise associated with mutations in *RAD50*.^4^ In both Japanese patients, variants of close-by nucleotides to the mutated nucleotide in our patients were found. One patient was compound heterozygote for c.658A>C and c.659+1G>A and the other patient was compound heterozygote c.658A>C and c.338A>G. The *MRE11* c.658A>C variant does not lead to alteration of amino acid (p.Arg220=) and the results from reverse-transcriptase PCR analysis in these patients carrying the transcript of the c.658A>C allele indicated that this variant leads to exon 7 skipping, but that some small amount of RNA was correctly spliced.^17^ Correspondingly, a reduced amount of wild-type–sized MRE11A protein was detected by immunoblot analysis.^17^ This indicates that c.657C>T alteration in our patients has a greater impact on splicing efficiency than the c.658A>C variant, leading to complete destabilization of the transcript by NMD and subsequent depletion of MRE11A.

**Table:**
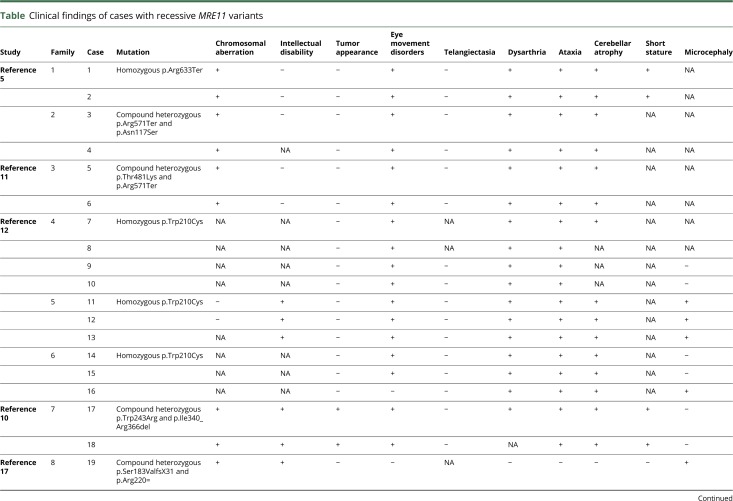

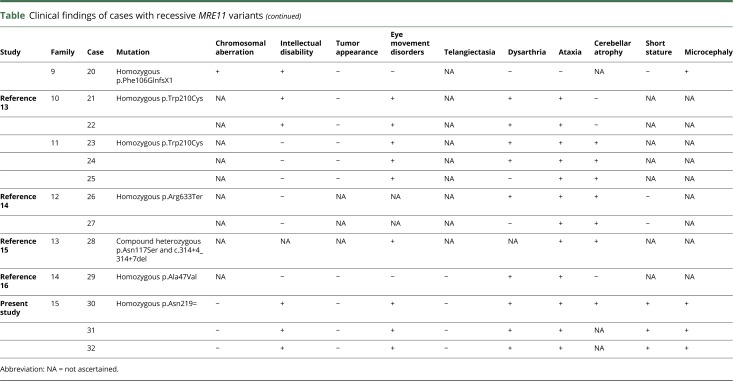
Clinical findings of cases with recessive *MRE11* variants

The MRN complex is involved in sensing of DNA double-strand breaks, DNA recombination, and multiple cell-cycle checkpoints.^18,19^ Cooperation between the ATM and MRN complexes is essential in the DNA damage response, which is not fully determined.^20^ MRE11A, a member of the MRN complex, is involved in homologous and mitotic and meiotic recombination, telomere length maintenance, and DNA double-strand break repair.^21^ MRE11A possesses DNA exonuclease and endonuclease activities that are highly conserved during evolution.^22^ The relatively mild impact of MRE11A deficiency, which permits viability in our patients, is intriguing. In addition, it is in sharp contrast to the early embryonic lethality of nuclease-deficient and null alleles of murine *Mre11*, associated with marked genome instability.^21,23^ However, despite the contribution of MRE11A in the MRN complex and the cooperation between the MRN complex and ATM in the DNA double-strand break repair pathways to maintain genomic integrity,^20^ loss of MRE11A only modestly impaired double-strand break repair in the chicken DT40 and human TK6 cell lines.^24^ Furthermore, animal models of NBS1 or MRE11A do not completely recapitulate phenotypes observed in NBS or ATLD patients, including the neurologic aspects.^25–27^

Notably, complete absence of ATM kinase, in patients with ataxia telangiectasia, a cancer-prone neurodegenerative disease, is not lethal.^28^ The majority of the variants in *ATM* in patients with classic ataxia telangiectasia are biallelic truncating mutations that result in a total loss of destabilized ATM protein.^28^ Given the vital role of ATM in the DNA damage response for DNA repair, cell cycle checkpoint activation, and apoptosis,^29^ the viability associated with loss of ATM in ataxia telangiectasia patients is intriguing. Mouse models deficient in ATM recapitulate accurate ataxia telangiectasia disease phenotypes,^30–32^ but loss of ATM kinase activity causes early embryonic lethality in mice, indicating that inhibition of ATM kinase activity does not equate to loss of the ATM protein.^33^ It was speculated that embryonic lethality in mice with ATM kinase inactivity was the result of ATM kinase recruitment at DNA breaks, which may impair the function of other proteins by blocking their access to DNA damage.^33^

Although MRE11A, NBS1, and RAD50 are components of the MRN complex, mutations in *MRE11*, *NBN*, and *RAD50* are associated with different clinical phenotypes, suggesting that the components have distinct functions and roles independent of the MRN complex. Patients with NBS and ataxia telangiectasia have a predisposition to cancer, particularly an increased risk of developing lymphoid tumors,^20^ which may reflect the involvement of ATM and MRN complex in DNA damage response. Nevertheless, development of cancer in ATLD patients associated with *MRE11* has not been a frequent finding in reported cases, with only 2 patients so far,^10^ and it is not a clinical feature in the family reported here. It thus remains unknown whether patients with ATLD have a predisposition to cancer, given the few patients with *MRE11* mutation that have been described so far.

Patients with ataxia telangiectasia, NBS, and ATLD usually show an increased level of chromosomal translocation in the peripheral blood involving chromosome 7 and 14.^20^ Homozygous *MRE11* variants often alter not only the levels of MRE11A but also the levels of 2 other components of the MRN complex, NBS1 and RAD50, leading to inactivation of the entire MRN protein complex.^5,9,11,17^ Although, the effect of *MRE11* c.657C>T variant on sensitivity to ionizing radiation and the impact on NBS1 and RAD50 expression levels or the entire MRN complex in our patients remain unknown, none of the homozygous or heterozygous carriers of the *MRE11* variant in our family show any chromosomal abnormalities. This is presumably because of retained contribution of damage sensor or mediators involved in the MRN complex and sustained ATM kinase activity in maintaining chromosomal integrity, as observed in other ATLD patients associated with *MRE11* mutation.^34^ However, absence of chromosomal alterations in this family is intriguing and requires further investigations.

We describe patients with characteristic features of ATLD, associated with a homozygous *MRE11* splicing variant leading to RNA decay and deficiency of MRE11A protein.

## Glossary

ATLD: ataxia-telangiectasia-like disorder
ATM: ataxia telangiectasia mutated
MMC: mitomycin C
NBS: Nijmegen breakage syndrome
NBSLD: Nijmegen breakage syndrome-like disorder
NGS: Next-generation sequencing
NMD: nonsense-mediated mRNA decay
NSES: neuromuscular sub-exomic sequencing
WES: whole exome sequencing
